# Climate change, pharmaceuticals and prescribing patterns: a scoping review and proposal for the MAMS model for healthcare educators

**DOI:** 10.3389/fpubh.2026.1806075

**Published:** 2026-08-24

**Authors:** Kenneth Mueller, Heather Allstrom, Nina Ali, Sharon Leslie, Lori Modly, Joseph Crane, Rebecca Philipsborn, Daniel Jackson Smith

**Affiliations:** 1 Nell Hodgson Woodruff School of Nursing, Emory University, Atlanta, GA, United States; 2 School of Nursing, University at Buffalo, Buffalo, NY, United States; 3 Woodruff Health Sciences Center Library, Emory University, Atlanta, GA, United States; 4 Department of Pediatrics, Emory University School of Medicine, Atlanta, GA, United States

**Keywords:** climate change, health professions education, scoping review, pharmaceuticals, pharmacology, prescribing patterns, sustainable healthcare, climate-conscious prescribing

## Abstract

**Background:**

Healthcare contributes substantially to climate change, and pharmaceuticals account for a meaningful share of this footprint across manufacturing, distribution, prescribing, and disposal. However, climate-health education and prescribing practices are often addressed separately, leaving limited guidance for integrating climate change, pharmaceuticals, and prescribing patterns within a single framework. This scoping review synthesizes evidence on the intersection of these domains, with attention to how these concepts are incorporated into health professions education (HPE) and clinical practice.

**Methods:**

A comprehensive literature search strategy of six databases (PubMed, Embase, CINAHL, ERIC, Health Source: Nursing Academic, and Web of Science) identified peer-reviewed studies published between January 1988 and December 2025 in English or Spanish. Eligible studies addressed climate change in relation to pharmaceutical use or prescribing patterns and included an educational or practice-based component; animal studies, studies without interventions, and non-prescribing studies were excluded. Searches were conducted in December 2023 and updated in April 2025. Data were extracted on study characteristics, clinical domain, educational approach, and directional relationships mapped to the MAMS model dyads (Climate ↔ pharmaceuticals; pharmaceuticals ↔ prescribing patterns; climate ↔ prescribing patterns).

**Results:**

From 4,247 citations, 28 studies met inclusion criteria. Studies were conducted across multiple regions, most commonly the United States (*n* = 6), United Kingdom (*n* = 5), Australia (*n* = 5), and Canada (*n* = 4), with additional single country and multinational collaborations. Clinical domains varied, with the largest representation in anesthesia and perioperative care (*n* = 8), followed by pharmacy and pharmacology (*n* = 5), primary care (*n* = 3), and fewer in other specialties. Studies used single and multi-educational domains, most commonly framework development (*n* = 16), followed by curricular integration (*n* = 6). Outcomes included reductions in anesthetic or inhaler related emissions, behavior change, knowledge or awareness, attitudes or satisfaction, and identification of barriers and facilitators. Although individual MAMS dyads were frequently examined, no study operationalized all three domains in an integrated bidirectional tri-dyadic manner.

**Conclusion:**

Existing literature on climate-conscious prescribing education is expanding but remains clinically concentrated and largely conceptual, with limited evaluation of instructional strategies. No study operationalized a fully bidirectional, tri-dyadic approach linking climate change, pharmaceuticals, and prescribing patterns, underscoring the need for integrated, outcomes-oriented frameworks to guide HPE and practice change.

## Introduction

The healthcare sector is a major contributor to climate change; if it were a country, it would rank fifth globally in greenhouse gas emissions (1). Pharmaceuticals account for a significant share of this footprint across their lifecycle, including manufacturing, distribution, prescribing, and disposal, with certain medications, such as inhalers, identified as high-impact contributors (2). Yet, discussions on climate and health often overlook pharmaceuticals and prescribing patterns. Existing research tends to address these domains in isolation (3) and in a unilateral direction, leaving a gap in understanding their intersection. Because prescribers play a central role in shaping pharmaceutical-related emissions (4), a clearer understanding of how climate change, pharmaceuticals, and prescribing patterns intersect is essential to inform health professions education (HPE) on these topics. One practical goal is to embed sustainability into clinical decision-making.

Integrating sustainability into healthcare relies heavily on HPE as a key mechanism for change. Climate change must be systematically embedded into HPE curricula to prepare graduates for climate-related health impacts and equip them for environmentally responsible practice (5–7). Institutions such as medical schools and teaching hospitals face growing pressure to embed climate and health literacy as a core responsibility (8). However, integration remains inconsistent. Specialty-specific studies reveal gaps: for example, 90.3% of U.S. emergency medicine programs lack climate content (9), and dental curricula rarely include sustainability despite positive attitudes toward its adoption (10).

Faculty and students alike support climate integration. Surveys show strong faculty endorsement, though many institutions limit content to electives due to structural barriers (11). Students value sustainability but report lacking actionable skills, creating a persistent value–action gap (12–14). Healthcare professionals also express readiness for change: anesthesiologists and prescribers in Scotland advocate for sustainability in clinical decision-making, though time and expertise remain obstacles (15, 16). Evidence suggests that education can drive measurable environmental benefits; for instance, targeted initiatives in French hospitals significantly reduced anesthetic-related emissions (17).

Global agreements reinforce the urgency of integrating climate education into HPE. The Paris Agreement identifies education as a key mechanism for climate action (6), while Intergovernmental Panel on Climate Change (IPCC) targets demand rapid emissions reductions to limit warming to 1.5 °C (18). Collectively, these findings underscore the need for coherent, skills-based curricula that bridge awareness and practice, preparing clinicians to deliver high-quality care while advancing planetary health. HPE organizations and professional societies continue to call for engagement in climate and health education (19, 20). Despite these calls, limited attention has been given to the intersection of climate change, pharmaceuticals, and prescribing patterns, which is essential for preparing clinicians to make environmentally responsible, evidence-aligned decisions in practice.

**Figure 1:**
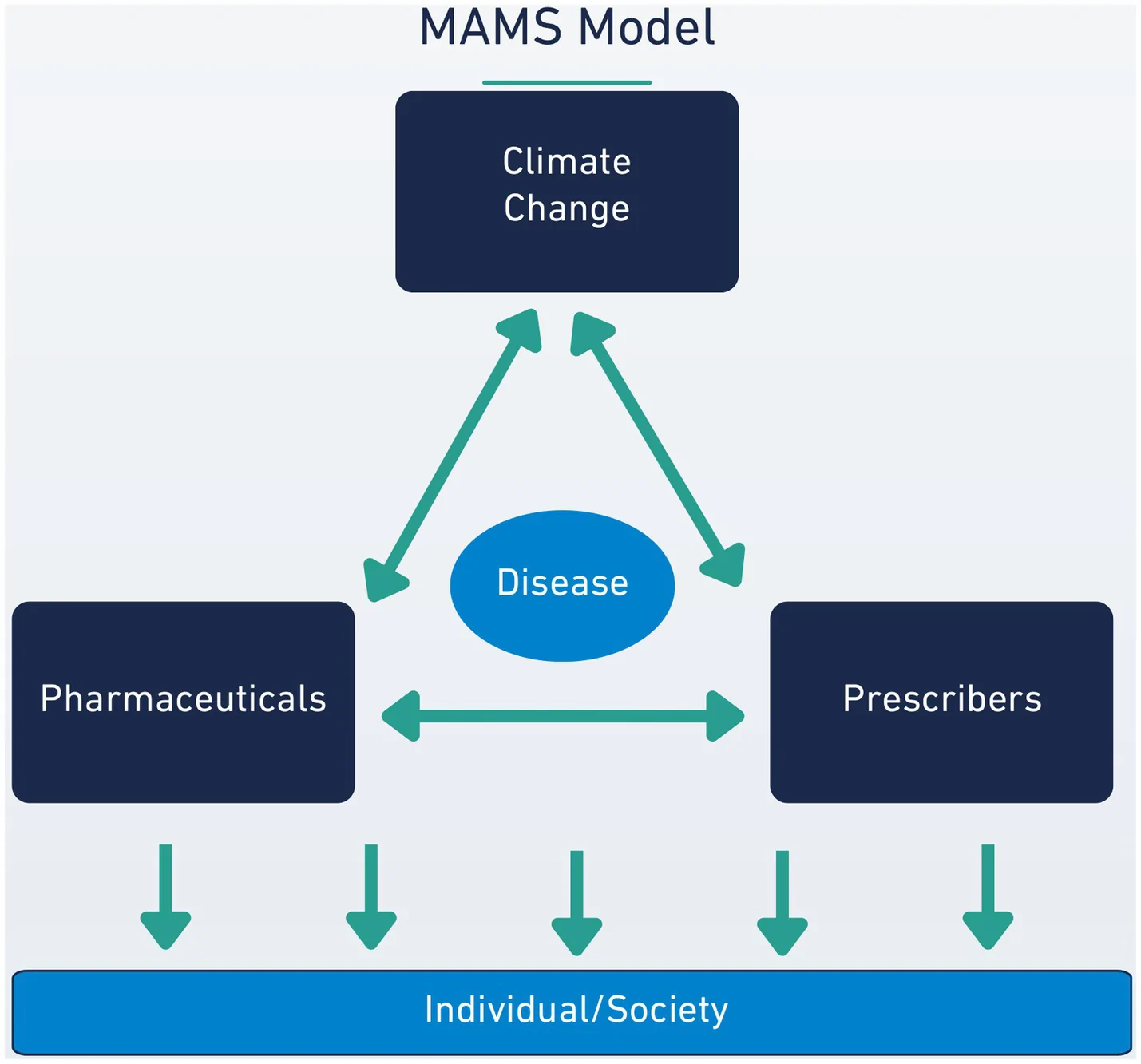
The MAMS model: a bidirectional, tri-dyadic conceptual framework linking Climate Change, Pharmaceuticals, and Prescribers/Prescribing Patterns, which together influence Disease and downstream Individual/Society outcomes. Each domain both influences and is influenced by the others, reflecting the interconnected pathways through which climate change, pharmaceutical use, and prescribing decisions shape health.

The primary authors previously published a paper on the integration of climate change content into an advanced pharmacology course for Advanced Practice Registered Nurses (APRNs) and developed a conceptual model to describe the bidirectional relationship of climate change, pharmaceuticals, and prescribing patterns that was theorized while exploring the literature (21). The conceptual model, named the MAMS model (Figure 1) after the initials of the primary authors (Kenneth Mueller, Heather Allstrom, Lori Modly, and Daniel Smith), integrates a bidirectional relationship amongst the three domains (MAMs domains) of climate change, pharmaceuticals, and prescribing patterns, creating a bidirectional, tri-dyadic conceptual framework. This conceptual model proves a useful tool to map the scope and characteristics of educational frameworks and interventions represented in the existing literature. Therefore, we conducted a scoping review to map the current evidence base to the MAMS model, to clarify how the intersection of climate change, pharmaceuticals and prescribing patterns are discussed within HPE, and to identify areas of overlap and gaps requiring further study. Specifically, we aim to address: (1) what is known about the relationship between climate change, pharmaceuticals, and prescribing patterns in healthcare, and (2) the extent to which these dyadic relationships are integrated into health professions education.

## Materials and methods

This review was organized and reported in accordance with the Preferred Reporting Items for Systematic Reviews and Meta-Analyses (PRISMA) Extension for Scoping Reviews (22). A scoping review was chosen to allow for inclusion of studies from any methodology type and for a broad exploration of the literature in the field. The team followed the Arksey and O’Malley framework for scoping reviews (23) which involves: (1) identifying the research question; (2) identifying relevant studies through a comprehensive search strategy; (3) selecting studies using predefined inclusion and exclusion criteria (Appendix A); (4) charting the data using a standardized extraction approach; and (5) collating, summarizing, and reporting the results to map key concepts, evidence types, and knowledge gaps; the optional consultation step was not undertaken for this review.

**Figure 2:**
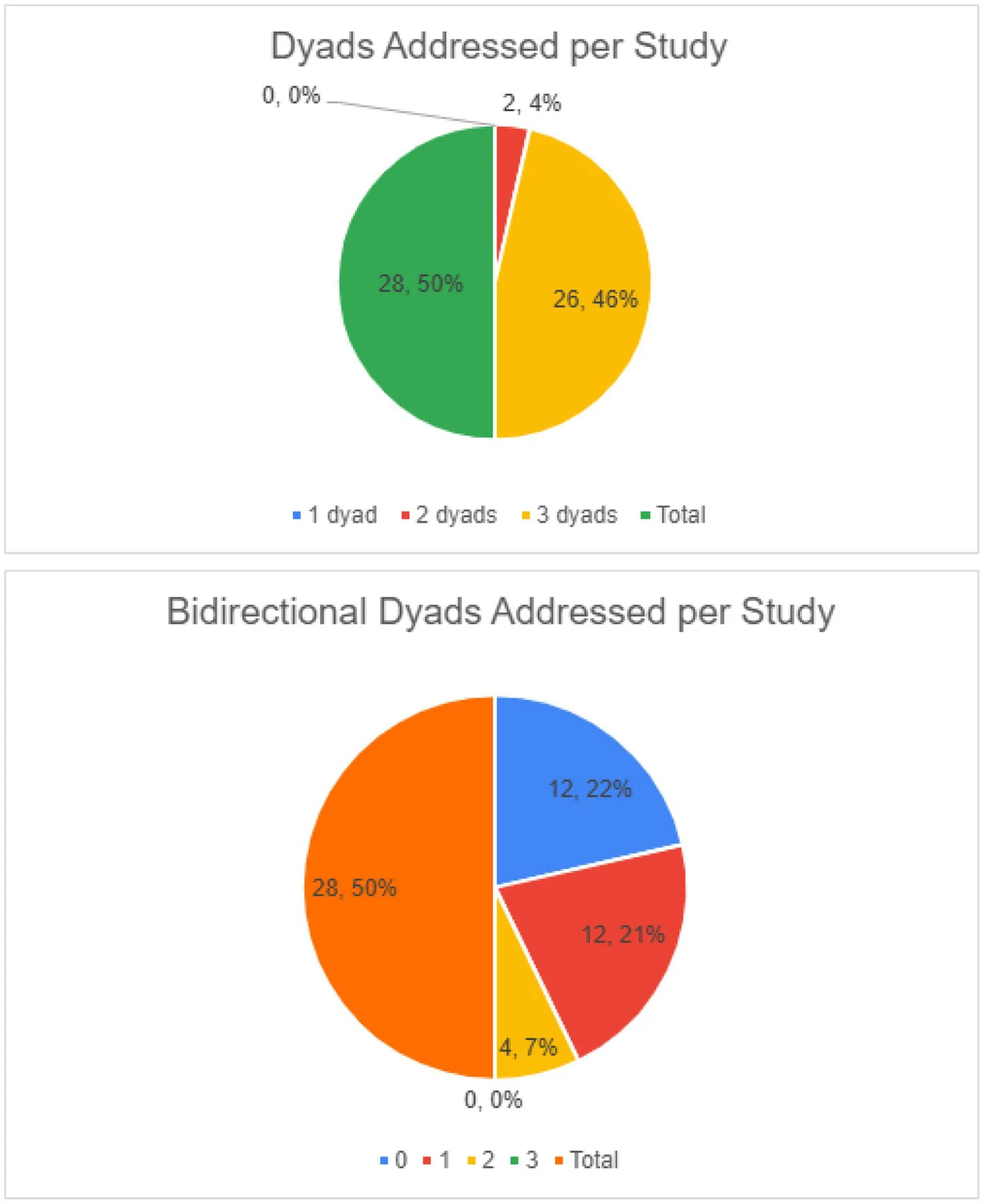
Distribution of MAMS model dyads addressed across the 28 included studies. (A) Total number of dyads (1, 2, or 3) addressed per study, regardless of relationship type. (B) Total number of bidirectional dyads (0, 1, 2, or 3) addressed per study, illustrating the extent to which studies captured the tri-dyadic, bidirectional nature of the MAMS model.

To develop Table 1, the team used a PRISMA-aligned data field within Covidence and employed a multirater screening and extraction process. For columns Education Framework/Model and Study Design, the team utilized agreed-upon operational definitions to categorize study design and educational model/frameworks of the studies; generative AI contributed to the initial drafting of the education domain definitions, as detailed in Appendix A. Many studies were determined to have multiple domains for healthcare specialty/target audience, primary clinical domain, and educational model/framework as further discussed in the results. Table 2 categorizes each study according to the extent of the relationship amongst each of the three MAMS domains (unidirectional, bidirectional or no relationship) and was completed by an individual reviewer. Ten percent of the entries were independently checked by a second reviewer, who confirmed initial categorizations. Figure 2 includes a total count of the number of dyads (1, 2, or 3) addressed within each study, regardless of relationship type, and Figure 2 also includes the total count of bidirectional relationship dyads (0, 1, 2, or 3) within each study to compare to the tri-dyadic nature of the MAMS model.

**Table 1:** Included studies.

Title	First author	Year of publication	Country in which the study conducted	Healthcare specialty/target audience	Primary clinical domain	Education model/framework	Study design	Results of the article
Sustainable medicines use in clinical practice: A clinical pharmacological view on eco-pharmaco-stewardship	Adeyeye et al. (45)	2022	UK	Pharmacists, Healthcare Providers, Anesthesia, Health systems administrators and policymakers	Anesthesia, perioperative care	Framework (theoretical/development/structure [development of content])	Literature Review - narrative	The article outlines major pharmaceutical and clinical contributors to healthcare emissions, with anesthetic gases and MDIs as key sources. Recommended strategies include lower-carbon anesthetic techniques, switching from MDIs when appropriate, optimizing prescribing, and improving drug manufacturing, packaging, and transport. It also emphasizes guideline changes, reducing waste through appropriate prescribing and adherence, and promoting safe disposal and recycling.
Modelling pharmacy and pharmaceutical students’ intentions to learn about their roles in environmental sustainability	Arslan et al. (41)	2024	UK	Pharmacy & Pharmaceutical sciences students	Pharmacy, pharmacology, prescribing	Framework (theoretical/development/structure [development of content])	Cross sectional study	Survey of 184 pharmacy students identified a five-factor model (environmental concern, attitudes, subjective norms, perceived behavioral control, and intentions) describing students’ intentions to learn about their roles in sustainability (LARS).
Climate change for the pulmonologist: a focused review	Balakrishnan et al. (49)	2023	USA	All health care providers (clinical, pulmonary emphasis)	Pulmonology, respiratory care	Framework (theoretical/development/structure [development of content])	Literature review	Climate change and air pollution worsen a wide range of conditions like asthma, COPD, infections, ILD, pulmonary hypertension, lung cancer, and sleep disorders. And increases risks in critical care and lung transplantation. The article highlights heightened vulnerability in children and certain occupations. It emphasizes that pulmonologists should counsel patients on exposure reduction and support low-carbon practices, policy action, and healthcare system mitigation efforts.
Education for the anthropocene: planetary health, sustainable health care, and the health workforce	Barna et al. (38)	2020	UK, Norway, India, Canada, The Netherlands	Health Profession Education (HPE)	Medical education, health professions education	Curricula (integration into school/university for a program/course [learner + instructor roles])	Conceptual framework	Recommends integrating environmental stewardship into health professions education through three domains: values (embedding sustainability and equity into professional ethics), knowledge (teaching planetary health and prevention of environmental health harms across curriculum), and skills (building clinical, leadership, advocacy, and research competencies that support sustainability).
Exploring anesthetists views on the carbon footprint of anesthesia and identifying opportunities and challenges for reducing its impact on the environment	Breth-Petersen et al. (44)	2024	Australia	Anesthesiology	Anesthesia, perioperative care	Other	Qualitative research	Interviews with 28 anesthetists revealed sevoflurane dominated practice, with low desflurane and nitrous oxide use. Using COM-B, barriers to lower-emission techniques included limited TIVA skills, time constraints, and habit, while motivation stemmed from patient safety and environmental responsibility. Recommended strategies included education, training, audit and feedback, departmental standards, and integrating sustainability into curricula.
Environmental impact and provider satisfaction associated with ePrescriptions in otolaryngology: a quality improvement study	Chandiramohan et al. (26)	2025	Canada	Otolaryngology (ENT)	Otolaryngology (ENT)	Curricula (integration into school/university for a program/course [learner + instructor roles])	Quasi-experimental study	Educational sessions, quarterly reporting, and recognition of prescribers increased ePrescription use in an Otolaryngology department from 9.7 to 40.7%, with pediatric ENT reaching 61.2%. The initiative yielded CO₂ savings at 125.9 lbs. and was well received by both providers and patients, who reported high satisfaction and minimal added burden.
Reducing the environmental impact of inhalers in primary care	Day (30)	2024	UK	Primary Care, Asthma	Primary care, general practice	Other	Literature review - narrative	pMDIs have much higher carbon impact than DPIs, and switching suitable patients can reduce emissions by up to 50% while maintaining asthma control. Avoiding high-GWP propellants and promoting recycling can further cut emissions, though recycling rates remain low. Education, patient engagement, and safe disposal are key to sustainable inhaler use.
The Gordon Wilson lecture: climate, health, and equity: the case for collective action from the health system	Dzau (42)	2024	USA	healthcare professionals, policy makers	Health systems, policy, leadership	Workshop (singular event)	Other: Lecture [of U.S. National Academy of Medicine efforts]	Healthcare must address climate change as a public health and equity crisis by reducing its carbon footprint and supporting resilient systems. NAM’s 2020 Grand Challenge outlines five steps: communicate urgency and educate professionals; guide system-wide transformation; accelerate research with community engagement; reduce climate-related inequities; and drive decarbonization in the health sector through practical tools and resources.
Environmental sustainability: an essential component of rational use of medicines	Giunchi et al. (46)	2024	Italy, Sweden	Pharmacology	Pharmacy, pharmacology, prescribing	Framework (theoretical/development/structure [development of content])	Conceptual framework	The article reviews the environmental impacts of medicines and proposes expanding the concept of rational medicine use to include One Health and sustainability principles. It presents a flowchart of upstream strategies (legislation, education, green drug design) and downstream strategies (proper disposal, wastewater treatment), alongside clinical actions such as rational prescribing, deprescribing, and sustainable dispensing.
Promoting climate change issues in medical education: Lessons from a student-driven advocacy project in a Canadian Medical School	Hansen et al. (35)	2021	Canada, Australia, Nigeria	Medical Students	Medical education, health professions education	Curricula (integration into school/university for a program/course [learner + instructor roles])	Case study	A student-led advocacy project at McMaster expanded climate–health education through guest lectures, an online series, and administrative collaboration that secured a temporary mandatory lecture. Students reported low baseline knowledge but high interest. The project produced learning resources and experiential activities. Recommendations include formal curricular integration and dedicated administrative support for sustainability in medical education.
Towards a climate-resilient primary health care service	Lokotola (47)	2023	South Africa	Primary care	Primary care, general practice	Other; framework (theoretical/development/structure [development of content])	Literature review - narrative	The article reviews how climate change impacts primary care and outlines adaptation strategies using the WHO climate-resilient health systems framework, emphasizing governance, workforce training, and emergency preparedness. It also highlights mitigation opportunities through the Global Green and Healthy Hospitals agenda, including carbon reduction, energy efficiency, waste management, and sustainable procurement. Family doctors are identified as key leaders for sustainability.
Why be sustainable? The Australian and New Zealand College of Anaesthetists Professional Document PS64: Statement on Environmental Sustainability in Anaesthesia and Pain Medicine Practice and its accompanying background paper	McGain et al. (27)	2019	Australia, New Zealand	Anesthesia, pain medicine	Anesthesia, perioperative care	Framework (theoretical/development/structure [development of content])	Case study	Advocates for sustainable anesthesia by avoiding high-GWP agents like desflurane and nitrous oxide, practicing low-flow anesthesia, and reusing resources in the OR. Emphasizes life-cycle assessments and need for ongoing research.
Reducing the carbon footprint of anesthesia: low-flow anesthesia and other techniques	McGowan (28)	2022	USA	CRNAs/Anesthesia	Anesthesia, perioperative care	Framework (theoretical/development/structure [development of content])	Literature review - narrative	Reduction of carbon footprint through decreasing use of inhaled volatile agents and switching to total intravenous anesthesia. Includes CO2 emissions comparison across agents like sevoflurane and desflurane.
Embedding planetary health concepts in a pre-medical physiology subject	Moro et al. (34)	2023	Australia	Pre-medical, physiology faculty and administrators	Medical education, health professions education	Curricula (integration into school/university for a program/course [learner + instructor roles])	Quasi-experimental study	After the intervention, 71% of students could define planetary health and 50% reported changes in their behaviors or thinking. Students valued learning about healthcare’s environmental impact, and the authors recommend adding a brief introductory overview (e.g., in the first lecture or a short video) to strengthen future teaching.
Characterization of an elective course on climate change for health professional students	Nguyen et al. (37)	2021	USA	Pharmacy, interprofessional health professional students, faculty, and administrators	Medical education, health professions education	Curricula (integration into school/university for a program/course [learner + instructor roles])	Cross-sectional study	Students rated the course highly for relevance and diverse perspectives and requested more actionable clinical examples. The program plans to evaluate knowledge retention and impacts on attitudes and professional behaviors.
An EAACI review: Go green in health care and research. Practical suggestions for sustainability in clinical practice, laboratories, and scientific meetings	Pali-Schöll et al. (48)	2023	Austria, Germany, Romania, USA	Healthcare professionals, biomedical labs	Research, labs, meetings	Framework (theoretical/development/structure [development of content])	Conceptual framework	Healthcare and research generate 4–10% of global GHG emissions. Recommended actions include energy-efficient labs, lower-carbon clinical practices (e.g., sustainable inhaler use), and virtual meetings. Key research gaps remain on the environmental footprint of drugs, treatments, diagnostics, and effective recycling strategies.
An EAACI review: Go green in health care and research. Practical suggestions for sustainability in clinical practice, laboratories, and scientific meetings How should institutions help clinicians to practice greener anaesthesia: first-order and second-order responsibilities to practice sustainably	Parker et al. (50)	2023	UK	Anesthesia practitioners, medical schools, hospital administrators	Anesthesia, perioperative care	Framework (theoretical/development/structure [development of content])	Conceptual framework	The article distinguishes second-order institutional responsibilities (who has responsibility) from first-order clinician responsibilities, such as banning high–GWP anesthetic gases. While restricting agents could drive practice change, it raises concerns about autonomy and situations where certain gases are clinically necessary. The authors highlight the need for consensus-building, education, and green nudges to support sustainable anesthesia practice.
Changing perioperative practice in the time of climate crisis	Pemberton et al. (29)	2023	Australia	Anesthesia and perioperative care	Anesthesia, perioperative care	Framework (theoretical/development/structure [development of content])	Quasi-experimental study	A survey of anesthetic staff showed that the intervention improved knowledge, particularly about nitrous oxide’s global-warming impact. Support for removing desflurane increased from 34 to 47%. Concerns about TIVA remained (plastic waste and water pollution). Overall, the education improved awareness but indicated that further strategies are needed before major practice changes can be implemented.
A pediatrician’s guide to climate change-informed primary care	Philipsborn et al. (60)	2021	USA	pediatrics, primary care	Pediatrics	Framework (theoretical/development/structure [development of content])	Other: practical guide	The framework provides pediatricians with structured, climate-informed guidance across routine primary care visits. The model includes practical screening questions, anticipatory guidance points, and intervention tools, aligned with AAP recommendations to address climate and health equity.
Climate change and the practice of medicine: essentials for resident education	Philipsborn et al. (39)	2021	USA	MD residents, medical students	Medical education, health professions education	Curricula (integration into school/university for a program/course [learner + instructor roles])	Conceptual framework	The article presents a climate-health education framework for medical residents, outlining vulnerable patient populations and trainee education. Core content includes climate-related impacts on heat illness, air quality, infectious diseases, nutrition, injuries, mental health, and displacement, along with adaptation strategies, disaster preparedness, sustainable healthcare delivery, and physician advocacy. The framework is adaptable across specialties.
Developing a behavior change intervention using information about greenhouse gas emissions to reduce liquid antibiotic prescribing	Pickles et al. (43)	2025	UK	Primary care and pharmacists	Primary care, general practice	Framework (theoretical/development/structure [development of content])	Qualitative research	Interviews with 11 primary care staff in North East England informed a behavior change intervention to reduce liquid antibiotic prescribing. Using the COM-B model, participants highlighted motivations (awareness of high liquid use, climate impact, pill-swallowing ability), opportunities (cost-saving initiatives, sustainability initiatives, past shortages), and capabilities (resources). Benchmarking and emissions data, and cost and shortage concerns were motivating. Most participants said they would share and use the resources to guide prescribing, despite barriers such as limited time and hierarchical dynamics.
12 tips for teaching environmental sustainability to health professionals	Schwerdtle et al. (40)	2020	Australia	Health care professionals	Medical education, health professions education	Framework (theoretical/development/structure [development of content])	Conceptual framework	The article outlines key principles for integrating climate and planetary health into health professions education. Recommendations include linking sustainability to core competencies, teaching adaptation and mitigation strategies, emphasizing health co-benefits, expanding professionalism to include environmental ethics and advocacy, prioritizing locally relevant content, and using reflective, formative assessment.
How ophthalmologists can decarbonize eye care: a review of existing sustainability strategies and steps ophthalmologists can take	Sherry et al. (31)	2023	USA	Ophthalmologists	Ophthalmology	Framework (theoretical/development/structure [development of content])	Literature review	The literature identifies several safe, low-cost, lower-emission surgical interventions, such as dispensing medications at home, multi-dosing, improved waste sorting, reducing surgical supplies, and use of immediate sequential bilateral cataract surgery. Evidence gaps remain around switching single-use items to reusables and adopting hub-and-spoke operating room setup.
A Scoping review of planetary health education in pharmacy curricula	Urslak et al. (36)	2025	Canada	Pharmacy	Pharmacy, pharmacology, prescribing	Framework (theoretical/development/structure [development of content])	Literature review	A review of 25 pharmacy schools across 12 countries found early, inconsistent integration of planetary health content, with most programs offering didactic lectures on environmental impacts and mitigation strategies. Teaching methods varied and few schools embedded content across multiple courses. Common barriers included limited faculty expertise and crowded curricula, while facilitators included faculty champions and student demand. Overall, pharmacy education lacks consensus on core competencies and requires more coordinated approaches.
Unwarranted variation and the goal of net zero for the NHS in England: exploring the link between efficiency working, patient outcomes and carbon footprint	van Hove et al. (32)	2024	UK	Anesthesia	Anesthesia, perioperative care	Framework (theoretical/development/structure [development of content])	Case study	Standardizing surgical pathways, such as pre-op assessment, virtual appointments, expanding day-case surgery, and reducing unnecessary procedures, can improve outcomes while cutting emissions. Modelling demonstrates substantial carbon savings, and the authors note key barriers (cultural resistance, limited data, infrastructure gaps) and facilitators (national guidance, best-practice pathways). Overall, embedding sustainability into GIRFT can drive lower-carbon surgical care but requires coordinated system-wide effort.
Developing a guide for sustainable healthcare practice: a case study from the Swedish Society of Medicine	Vilhelmsson et al. (33)	2025	Sweden	medical students and healthcare providers	Health systems, policy, leadership	Framework (theoretical/development/structure [development of content])	Case study	A Swedish sustainability guide was well received by clinicians as a practical tool for integrating greener practices, including reducing unnecessary tests, choosing lower-impact medications, and optimizing care delivery. It was incorporated into medical education, improving students’ sustainability knowledge, and inspired other health professions to develop similar tools.
Sustainability standards in pediatric anesthesia: quality initiative to reduce costly environmentally harmful volatile Anesthetics	Waberski et al. (25)	2023	USA	Pediatric anesthesiology	Anesthesia, perioperative care	Framework (theoretical/development/structure [development of content]); Curricula (integration into school/university for a program/course [learner + instructor roles])	Quasi-experimental study	The three-year QI initiative achieved a 77% reduction in anesthetic-related emissions by phasing out desflurane and promoting low-flow anesthesia. Provider education, system prompts, default low-flow settings, and restricted desflurane access drove the change. The project also reduced costs by 41%, showing that systems-level interventions can shift anesthesiology to more sustainable options.
ED inhaler revolution: a simple method to substantially reduce the carbon footprint and cost of inhaler use in the emergency department	Wilson et al. (24)	2025	Canada	Emergency Department clinicians	Emergency medicine	Other; Curricula (integration into school/university for a program/course [learner + instructor roles])	Quasi-experimental study	Passive methods (emails, posters) were followed by active on-shift sessions and simulations in this educational campaign. Over 12 months, MDI dispensations decreased by 19% in the first six months and 43% in the second six months, dry-powder inhaler use increased from 1 to 12 dispensations/month, and staff knowledge of inhaler carbon impact improved by 60%. Brief, in-person education was most effective.

Literature review using PRISMA-aligned data field with in covidence and employing a multirater screening and extraction process. For columns Education Framework/Model and Study Design, the team utilized agreed-upon operational definitions to categorize study design and educational model/frameworks of the studies; generative AI contributed to the initial drafting of the education domain definitions, as detailed in Appendix A. Many studies were determined to have multiple domains for healthcare specialty/target audience, primary clinical domain, and educational model/framework as further discussed in the results.

**Table 2:** Systematic review of directional relationships between climate, pharmaceuticals, and prescribing patterns (MAMS model).

First author (citation)	Climate ↔ pharmaceuticals	Pharmaceuticals ↔ prescribing patterns	Climate ↔ prescribing patterns
Adeyeye et al. (45)	**←**	**↔**	**←**
Arslan et al. (41)	**←**	**∅**	**←**
Balakrishnan et al. (49)	**←**	**→**	**↔**
Barna et al. (38)	**←**	**→**	**←**
Breth-Petersen et al. (44)	**←**	**→**	**→**
Chandiramohan et al. (26)	**←**	**→**	**←**
Day (30)	**↔**	**→**	**←**
Dzau and Laitner (42)	**←**	**↔**	**←**
Giunchi et al. (46)	**←**	**↔**	**←**
Hansen et al. (35)	**←**	**→**	**←**
Lokotola (47)	**←**	**∅**	**←**
McGain et al. (27)	**←**	**↔**	**←**
McGowan (28)	**←**	**→**	**←**
Moro et al. (34)	**←**	**←**	**←**
Nguyen et al. (37)	**←**	**→**	**←**
Pali-Schöll et al. (48)	**←**	**↔**	**↔**
Parker et al. (50)	**←**	**→**	**←**
Pemberton et al. (29)	**←**	**↔**	**←**
Philipsborn et al. (60)	**↔**	**→**	**↔**
Philipsborn et al. (39)	**↔**	**→**	**←**
Pickles et al. (43)	**←**	**→**	**←**
Schwerdtle et al. (40)	**←**	**←**	**↔**
Sherry et al. (31)	**←**	**↔**	**↔**
Urslak et al. (36)	**↔**	**↔**	**←**
van Hove et al. (32)	**←**	**→**	**←**
Vilhelmsson et al. (33)	**←**	**↔**	**←**
Waberski et al. (25)	**←**	**↔**	**←**
Wilson et al. (24)	**←**	**↔**	**←**

This table presents a comprehensive analysis of 28 peer-reviewed studies published between 2019 and 2025, examining the directional relationships in the MAMS model; climate factors, pharmaceutical production/distribution, and prescribing patterns. Each study is categorized by the nature of relationships identified: unidirectional influences (indicated by directional arrows showing the direction of effect), bidirectional interactions (showing mutual influence), or absence of identified relationships. The synthesis reveals predominant patterns of climate impacts on pharmaceutical systems and subsequent effects on prescribing behaviors, with notable variations in bidirectional dynamics across different research contexts.

### Inclusion/exclusion criteria

Studies considered eligible were peer-reviewed articles published between January 1, 1988 and April 1, 2025 in English or Spanish, reporting on educational interventions or proposed interventions on educating healthcare students on the impacts of climate change and prescribing. The year 1988 was chosen as a starting date because it was the first meeting of the IPCC created by the World Meteorological Organization and the Nations Environment Programme (18). Studies were excluded if they had no intervention/proposed intervention, were animal studies, or were related to heat injury or cooling interventions in athletes or sports (Appendix A).

### Literature search strategy

Following the second step of the Arksey and O’Malley framework, a comprehensive literature search strategy was developed and conducted by an experienced medical librarian with input from the research team to identify relevant articles. Pre-identified sentinel articles were hand searched for keywords relating to the study objectives. An initial search strategy was tested in PubMed, results were assessed, and additional terms were culled from titles, abstracts, and subject headings. The searches supplemented controlled vocabulary with keywords related to health care professionals (e.g., pharmacist, clinician), pharmaceuticals (e.g., pharmacy, pharmaceutical preparations), education (e.g., curriculum, framework), and climate change (e.g., greenhouse gases, global warming). A second draft search with the added terms and proximity operators was again tested in PubMed. The strategy was reviewed by another medical librarian and then translated and executed across additional databases. Six bibliographic databases were searched: CINAHL (EBSCOhost), Embase.com (Elsevier), ERIC (EBSCOhost), Health Source: Nursing Academic (EBSCOhost), PubMed, and Web of Science Core Collection (Clarivate). Searches were initially undertaken December 1, 2023 and again April 1, 2025. Full search strategies for each database may be found in Appendix B.

## Results

### Study selection

**Figure 3:**
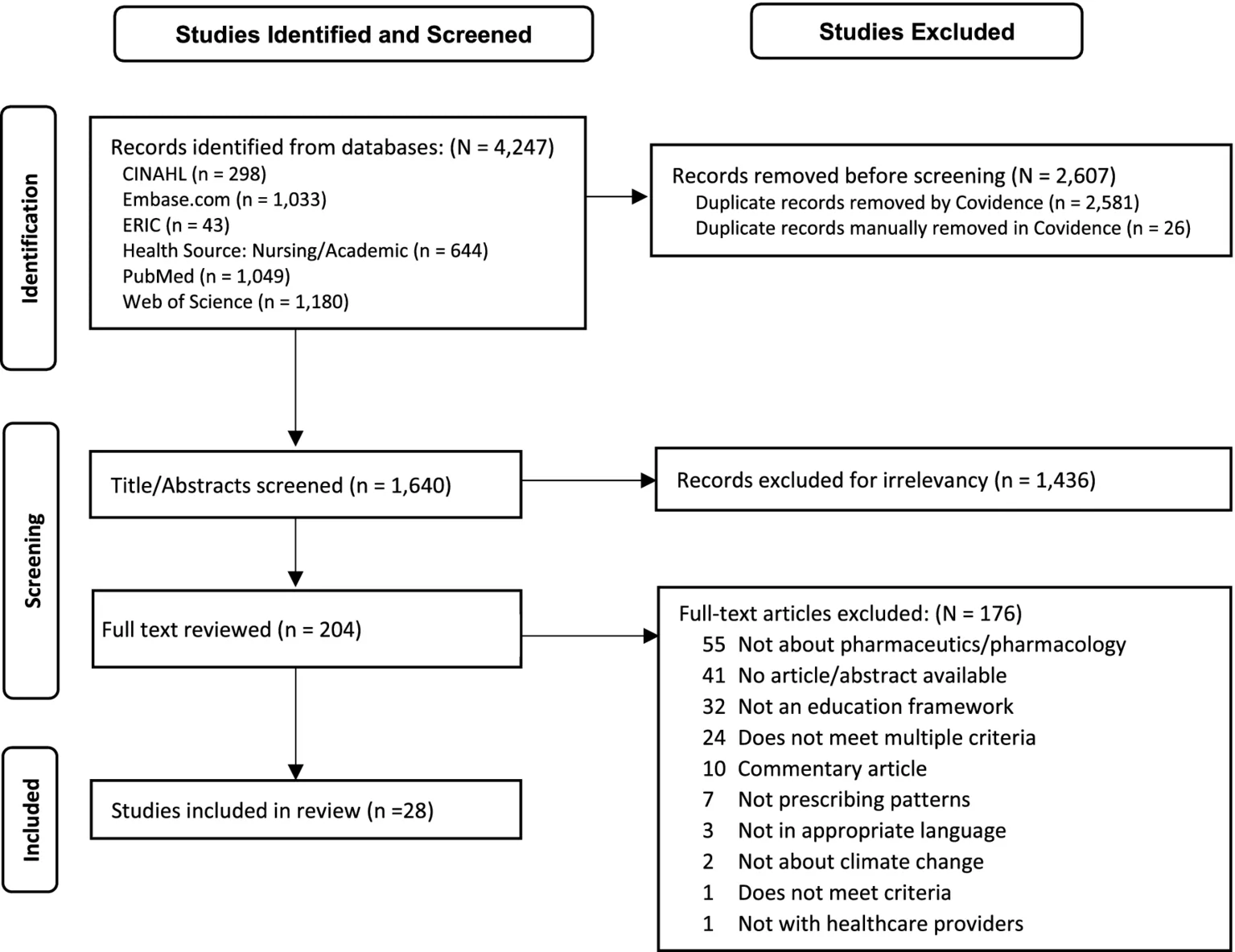
Adapted PRISMA flow diagram.

A total of 4,247 citations from the databases were uploaded to EndNote X20. These citations were then uploaded to Covidence systematic review software, which identified 2,581 duplicates, leaving 1,666 records. Title and abstract screening for eligibility according to the inclusion/exclusion criteria were performed by five independent investigators (Kenneth Mueller, Heather Allstrom, Nina Ali, Lori Modly, and Daniel Smith; Appendix A). Conflicts between the reviewers were resolved by discussion. If no consensus could be reached, the senior-author of this paper served as the final decision maker. A further 26 duplicates were identified by the reviewers and removed manually. Of the remaining records, 1,436 were excluded for irrelevancy, leaving 204 eligible for full-text review by four independent investigators (Kenneth Mueller, Heather Allstrom, Nina Ali, and Daniel Smith). Of these, 176 were excluded, leaving 28 for data extraction and synthesis. The review and selection processes for the studies are summarized in the diagram in Figure 3.

### Characteristics of sources of evidence

Publications that were included ranged from 2019 to 2025, with an upward trend in recent years. Studies were conducted across multiple regions. Most were carried out in a single country, most commonly the United States (*n* = 6), the United Kingdom (UK) (*n* = 5), Australia (*n* = 5), and Canada (*n* = 4). Additionally single-country studies were conducted in South Africa (*n* = 1) and Sweden (*n* = 1). Five studies were multinational, involving combinations of countries such as the UK, Norway, India, Canada, and the Netherlands; Italy and Sweden; Canada, Australia, and Nigeria; Australia and New Zealand; and Austria, Germany, Romania, and the United States. Clinical domains varied, with the largest representation in anesthesia and perioperative care (*n* = 8), followed by pharmacy and pharmacology (*n* = 5), primary care (*n* = 3), and smaller numbers in otolaryngology, ophthalmology, emergency medicine, and medical education. Most studies utilized a single educational domain, with the majority using framework development (*n* = 16), followed by curricular integration (*n* = 6), other approaches (*n* = 2), and one workshop-based intervention (*n* = 1). Three studies used multidomain educational approaches, combining multiple categories (*n* = 3). Study designs ranged widely, including narrative and other literature reviews (*n* = 7), conceptual frameworks (*n* = 6), quasi-experimental studies (*n* = 5), case studies (*n* = 4), and other designs such as qualitative research (*n* = 2), cross-sectional studies (*n* = 2), and practical guides or lectures (*n* = 2) (Table 1).

### Critical appraisal within sources of evidence

Formal critical appraisal was not conducted, as this review aimed to map the scope and characteristics of educational frameworks and interventions rather than assess methodological quality.

### Results of evidence

The included studies varied widely in scope, intervention type, and outcomes however, consistent thematic patterns emerged across five domains: (1) environmental impact reduction through practice change, (2) educational interventions and curricular integration, (3) knowledge, attitudes, and behavioral intentions, (4) barriers and facilitators to sustainable practice, and (5) conceptual and system-level frameworks. Several interventions combining education with system-level changes consistently demonstrated reductions in healthcare-related greenhouse gas emissions. Wilson et al. (24) reported decreased metered-dose inhaler (MDI) use decreased by 19% in the first 6 months and 43% in the second and increased dry-powder inhaler (DPI) adoption following a two-stage educational campaign, alongside improved staff knowledge. Similarly, Waberski et al. (25) achieved a 77% reduction in anesthetic emissions and 41% cost savings through desflurane phase-out and low-flow techniques, while Chandiramohan et al. (26) increased e-prescribing rates from 9.7 to 40.7%, with pediatric ENT reaching 61.2% with estimated CO₂ savings of 125.9 lbs. and high provider and patient satisfaction. Additional studies supported these findings across specialties: McGain (27), McGowan (28), and Pemberton (29) highlighted reductions in anesthetic emissions through agent selection and technique changes; Day (30) demonstrated that inhaler switching in primary care can substantially reduce emissions; and Sherry (31) identified lower-waste surgical practices in ophthalmology. At the systems level, van Hove et al. (32) and Vilhelmsson et al. (33) showed that standardizing care pathways and reducing unnecessary care can improve outcomes while lowering emissions. Overall, the greatest impact was observed when education was paired with structural or policy changes.

Educational interventions demonstrated consistent improvements in knowledge, awareness, and engagement, though integration across curricula remained variable. Moro et al. (34) embedded planetary health concepts into a pre-medical physiology course, with 71% of students able to define planetary health and its relevance to healthcare practice and 50% reporting changes in behaviors or thinking, particularly greater awareness of healthcare’s environmental impact and personal responsibility. Students also reported valuing content that highlighted the environmental impact of healthcare practices. The authors additionally recommended incorporating a brief introductory overview early in the course to reinforce learning. Hansen et al. (35) documented a student-led advocacy project that introduced climate-health education through guest lectures, an online learning series, and student developed learning resources with students reporting low baseline knowledge but high interest in climate-related topics. The initiative also resulted in the introduction of a temporary mandatory lecture within the medical curriculum and generated experiential learning opportunities, which highlights the need for sustained curricular integration and institutional support. Pharmacy-focused studies, such as Urslak et al. (36), revealed early but inconsistent integration of planetary health content across curricula globally, with most programs relying on didactic lectures and few embedding content longitudinally. Additional curricular initiatives reinforced these findings. Nguyen et al. (37) described an interprofessional elective course that was highly rated by learners for its relevance and breadth, although students requested more clinically actionable content. Conceptual frameworks further supported integration strategies; Barna et al. (38), Philipsborn et al. (39), and Schwerdtle et al. (40) emphasized embedding sustainability into core competencies, including values, clinical knowledge, advocacy, and systems-based practice. Overall, while educational interventions effectively increased awareness and engagement, most programs remained fragmented, with limited longitudinal integration or standardized competencies.

Several studies specifically examined changes in knowledge, attitudes, and behavioral intentions related to sustainable healthcare. Educational interventions consistently demonstrated improved awareness of healthcare’s environmental impact, as seen in studies by Moro et al. (34), Wilson et al. (24), and Pemberton et al. (29). Learners and clinicians frequently reported low baseline knowledge but strong interest in climate-related content, as highlighted by Hansen et al. (35) and Nguyen et al. (37). Behavioral intention was further explored by Arslan et al. (41), who identified key predictors of students’ willingness to engage in sustainability learning, including environmental concern, perceived social norms, and self-efficacy. At a broader level, Dzau and Laitner (42) emphasized the importance of framing climate change as a professional and ethical responsibility, highlighting the role of health system leadership in shaping attitudes and action. Together, these findings indicate that while knowledge gaps persist, there is strong motivation among learners and clinicians to engage in sustainable practices when provided with appropriate education and institutional support.

Qualitative research illuminated barriers and facilitators to sustainable prescribing. For example, Pickles et al. (43) used the Capability, Opportunity, Motivation-Behavior (COM-B) model to explore motivations and constraints among primary care staff, identifying benchmarking data, cost savings, and climate messaging as key drivers, while time limitations and hierarchical dynamics posed challenges. Breth-Petersen et al. (44) found that anesthetists acknowledged environmental responsibility but cited limited skills and entrenched habits as obstacles to adopting low-emission techniques. Common barriers identified across studies included limited time, entrenched practice patterns, lack of training, and hierarchical or institutional constraints. Facilitators included access to benchmarking data, cost-saving incentives, educational interventions, and system-level supports such as guidelines and default prescribing options. These findings underscore that knowledge alone is insufficient to change practice; instead, successful implementation requires alignment of individual capability, organizational opportunity, and motivational drivers.

A substantial proportion of included studies focused on conceptual frameworks and system-level strategies for integrating sustainability into healthcare. In pharmacology and prescribing, Adeyeye (45) and Giunchi et al. (46) emphasized eco-pharmacovigilance and rational prescribing practices that incorporate environmental considerations across the medication lifecycle. Broader system-level approaches were described by Lokotola et al. (47), who outlined climate-resilient primary care strategies, and Pali-Schöll et al. (48), who highlighted sustainability opportunities in clinical care, research, and healthcare operations. Specialty-specific frameworks, such as those by Balakrishnan et al. (49) and Parker et al. (50), emphasized the responsibility of clinicians and institutions to adopt lower-carbon practices while balancing clinical autonomy and patient care. Additionally, Dzau and Laitner (42) and Vilhelmsson et al. (33) highlighted the importance of leadership, policy, and practical guidance in advancing sustainable healthcare practices. While these frameworks provide comprehensive guidance across clinical, educational, and policy domains, few studies operationalized these concepts into fully integrated interventions, highlighting an ongoing gap between theory and practice.

## Synthesis of results

### Response data

**Figure 4:**
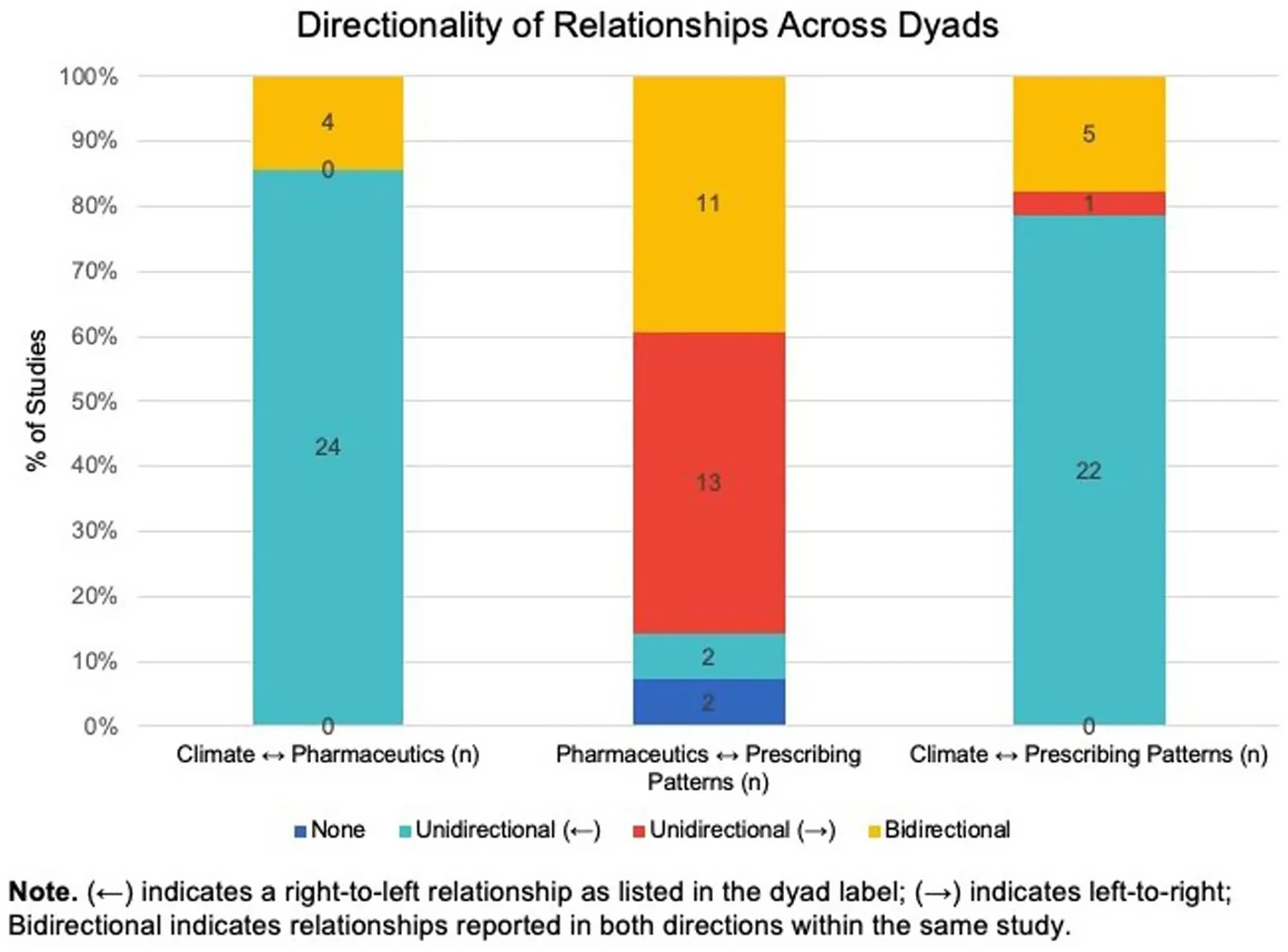
Directionality of relationships across the three MAMS model dyads (Climate–Pharmaceuticals, Pharmaceuticals–Prescribing Patterns, Climate–Prescribing Patterns) among the 28 included studies, categorized as no relationship, unidirectional (in either direction), or bidirectional, as coded in Table 2. The chart highlights that while individual dyads were frequently addressed, no study captured all three relationships in a fully bidirectional, tri-dyadic manner.

Across the 28 studies, outcomes clustered into five categories: environmental impact, behavior change, knowledge and awareness, attitudes and satisfaction, and barriers/facilitators. When each study was mapped to the MAMS model (Table 2), a clear gap emerged. All studies addressed at least one dyad (Climate Change ↔ Pharmaceuticals, Climate Change ↔ Prescribing Patterns, Prescribing Patterns↔ Pharmaceuticals), and 92.86% (*n* = 26) incorporated all three dyads to some degree. The two most common dyads were Climate Change ↔ Pharmaceuticals and Climate Change ↔ Prescribing Patterns (*n* = 28 each). The most frequently identified unidirectional dyad was the linear impact of pharmaceuticals on the climate (*n* = 24; Figure 4). 57.1% (*n* = 16) of the studies operationalized at least one bidirectional relationship (Figure 2). The most commonly identified bidirectional relationship within a dyad was Prescribing Patterns ↔ Pharmaceuticals (*n* = 11; Figure 4). Most importantly, none of the studies demonstrated a fully bidirectional, tri-dyadic approach and instead operationalized individual or partially connected dyads (Figure 2). This highlights a key opportunity for future curricula and conceptual models to more holistically connect climate, pharmaceuticals, and prescribing patterns in an integrated, bidirectional, and tri-dyadic manner.

## Discussion

Twenty-eight studies published between 2019 and 2025 addressed the intersections of climate change, pharmaceuticals, and prescribing patterns through various educational modalities. Collectively, these studies reflect a growing interest in environmentally sustainable healthcare; yet they also revealed a significant gap in connecting the three MAMS domains within HPE. A recent increase of publications shows trends toward climate-conscious prescribing, particularly within high-impact clinical specialties such as anesthesia, pharmacy, and primary care. However, the educational approach and implementation of the three MAMS domains, even in more recent literature, remains fragmented. Importantly, none of the studies operationalized a comprehensive, bidirectional tri-dyadic approach linking climate change, pharmaceuticals, and prescribing patterns, confirming the gap the MAMS model aims to address. This pattern, along with the other calls for a comprehensive analysis of health care emissions, demonstrates a need for a unified framework that provides a comprehensive understanding of climate-conscious prescribing and pharmaceutical impacts that can be embedded within HPE.

Across the included studies, several patterns emerged including study geographic origin, clinical concentration, education approach, and outcome type. First, studies were predominately from high income countries including the United State and United Kingdom. This aligns with current literature which shows substantial growth of climate-change education in HPE in high-income countries, while low and middle-income countries are behind (51). It is imperative that HPE regarding environmentally sustainable prescribing is a unified global effort, and this awareness provides an opportunity for expanded global engagement. Second, clinical concentrations from the studies clustered around high-impact specialties, such as anesthesia/peri-operative care, pharmacy/pharmacology, primary care, and pediatrics, with smaller representation from emergency medicine, ophthalmology, pulmonology, and otolaryngology. This distribution likely aligns with the professions’ use and growing awareness of high impact pharmaceutical carbon footprint agents such as volatile anesthetic gases utilized in the operating room and pMDI inhalers (52, 53). This clustering may also reflect an uneven distribution of climate-conscious prescribing education across healthcare professions and additionally may serve as an opportunity to expand curricular and research opportunities into underrepresented specialties. Third, that the education modalities used in the studies focused on conceptual framework development, rather than applied instruction, reflects the relative youth of this field. Among those that described educational interventions and their evaluations, approaches such as curricular integration, workshops, and mixed-methods interventions were used and generally tailored to specific learner groups.

Finally, highlighted outcomes across studies were dispersed amongst various categories including topics such as environmental impact, behavior change, knowledge gain, attitudes and satisfaction, and recognition of barriers and facilitators. Some interventions observed direct reductions in prescribing through a combination of passive and active educational modalities (24), while others saw reductions when educational modalities were paired with policy and systems-level interventions such as limited formularies/restricted access, and system prompts (25). Collectively, these findings reinforce the need that climate conscious prescribing should be inserted into all facets of HPE (2). Many studies used a mix of educational modalities and reported positive outcomes regardless of which MAMs domain dyad they addressed (29, 44, 50). However, none of the studies engaged all three dyads in a fully bidirectional manner. This limitation reflects a broader pattern: while progress is being made, current interventions do not capture the dynamic interplay across the entire tri-dyadic structure.

Recognizing this gap underscores the potential value of the MAMS model. If single- or dual-dyad interventions can already generate meaningful improvements in knowledge, attitudes, and even prescribing behaviors, the impact of a fully integrated, tri-dyadic framework is likely to be substantial, particularly when accounting for persistent barriers related to system structures, curricular implementation, and institutional priorities. The MAMS model should therefore be considered not only as a conceptual framework but also as a guiding structure for curricular design and evaluation. Such strategies may include longitudinal embedding of climate-conscious prescribing content across HPE curricula; layered instruction through didactic, experiential, and simulation-based training for learners; ongoing continuing education and point-of-care training for practicing clinicians; and the incorporation of systems-level policies that reinforce sustainable prescribing practices across the healthcare workforce.

### Addressing the gap: the MAMS model

The MAMS model (Figure 1), could address the lack of connection between dyads and support future educational efforts at the intersection of climate change, pharmaceuticals, and prescribing patterns. Each dyad has the potential for a bidirectional relationship amongst one another, and collectively, the model creates a bidirectional tri-dyadic conceptual framework. The MAMS model provides a comprehensive design of the complex yet interconnected relationships among these domains.

In the first domain (climate change ↔ pharmaceuticals), climate change directly affects medication stability, supply chains, and disposal pathways (54), while pharmaceuticals contribute to greenhouse gas emissions and environmental contamination across their lifecycle (55), illustrating how climate and medicines mutually shape one another. In the second domain (pharmaceuticals ↔ prescribing patterns), awareness of pharmaceuticals’ climate impacts can shift prescribing through clinician behavior, patient demand, and industry response (56, 57); for example, education about the footprint of pressurized metered-dose inhalers (pMDIs) has supported greater use of dry powder inhalers (DPIs), alongside manufacturer efforts to develop lower–global warming potential propellants for pMDIs (58). In the third domain (prescribing patterns ↔ climate change), climate-driven changes in disease burden influence prescribing (e.g., increased respiratory morbidity and inhaler use) (59), and prescribing patterns in turn affect climate impact through changes in medication volume and product mix used to manage these conditions. The impact of the specific pathology viewed through the three domains then converges to have impacts on the health outcomes at both the individual and societal levels. The MAMS model structure enables health professions programs to integrate all components of the framework into their curricula to develop a robust understanding of how each domain influences the others. This approach provides a streamlined opportunity for healthcare educators to apply some or all of the framework as it applies within their own curriculum and allow for complete core curriculum or elective course integration.

### Limitations

This review focused on HCP’s with prescriptive authority, which was appropriate given the emphasis on prescribing patterns. However, non-prescriptive HCPs, such as nurses, also play vital roles in medication management, patient education, and sustainability initiatives. Exclusion of non-prescriptive HCPs may unintentionally limit the importance and scope of interprofessional teams and their role on the bidirectional relationships of climate change, pharmaceuticals, and prescribing patterns. Additionally, the inclusion of only English and Spanish publications may have resulted in the omission of relevant studies from regions who are significantly impacted by healthcare related climate change. Climate change and sustainable practice are global priorities and important work published in other languages may not have been captured.

Our review also focused exclusively on peer-reviewed literature based on our search criteria, and may not have captured grey literature such as frameworks, educations, tool-kits, or other initiatives published by organizations such as the Planetary Health Alliance or World Health Organization (WHO). As a result, widely used, but non-peer reviewed education models may have been excluded. Although several studies discussed or incorporated external models such as One Health within their own frameworks or interventions (46), this review may underrepresent the availability of other tools and models available to educate HCPs. Additionally, some interventions included incorporation of sustainable healthcare within broader frameworks such as Getting It Right First Time (GIRFT), a national initiative for United Kingdom’s National Health Service (NHS) (32), recognizing that this discussion may be adaptable to non–direct climate change models (32). The MAMS model presented in this paper helps address this gap in a streamlined manner but does not replace the need for future reviews that examine organizational frameworks directly nor future educational interventions and their evaluations.

Finally, limitations may have occurred within the mapping of studies to the MAMS domains. Studies were diverse in their clinical specialty, educational modalities, study designs, outcomes, and chosen topics related to sustainable healthcare. MAMS domains were sometimes direct focal points within studies, and other times were mentioned briefly through indirect statements. Given this variability it is possible that domain mapping could vary.

## Conclusion

This scoping review mapped an emerging but fragmented body of literature at the intersection of climate change, pharmaceuticals, and prescribing patterns. Across 28 included studies, four consistent patterns were identified. First, the evidence base was clinically concentrated, with the strongest representation in anesthesia and perioperative care, suggesting that high-impact medication environments are driving much of the early innovation and measurement. Second, educational approaches were dominated by framework development and conceptual work, with fewer studies implementing longitudinal, competency-based curricula or evaluating sustained practice change over time. Third, outcomes were most commonly reported as measurable environmental impact, clinician behavior change, and knowledge or attitude shifts, reinforcing that targeted education paired with system-level supports, such as prompts, feedback, defaults, and access restrictions, can produce meaningful reductions in emissions without compromising care. Fourth, and most importantly, the literature remained largely dyad-focused and often linear or non-comprehensive. While many studies addressed at least one dyadic relationship, none operationalized a fully integrated, bidirectional tri-dyadic approach that simultaneously links climate change, pharmaceuticals, and prescribing patterns.

This scoping review highlights a critical gap for health professions education. When sustainability content is taught as isolated topic areas, learners may build awareness without gaining the practical, systems-informed competencies needed to translate that awareness into routine prescribing decisions across settings and disease states. To address this gap, we propose the MAMS model, a tri-dyadic conceptual framework that links climate change, pharmaceuticals, and prescribing patterns through bidirectional relationships. The MAMS model offers a structured foundation for curriculum design, interprofessional teaching, and evaluation planning by clarifying where educational content, clinical decision-making, and systems interventions intersect. It also supports outcomes-oriented implementation, including measurable environmental impact alongside traditional indicators of quality, safety, and patient-centered care.

Future work should prioritize implementation and evaluation of the MAMS model across disciplines, settings, and levels of training, including pharmacy, primary care, pediatrics, emergency medicine, and specialty practices beyond anesthesia. Studies should emphasize longitudinal competency development, assess durability of prescribing behavior change, and incorporate system-level levers that enable climate-conscious prescribing in real-world workflows. Expanding scope to better reflect interprofessional medication management and to include diverse geographic and linguistic contexts will further strengthen relevance and generalizability. By moving from isolated dyads to an integrated, bidirectional tri-dyadic perspective, health professions education can better prepare clinicians to deliver evidence-aligned, climate-conscious prescribing that advances both planetary health and patient outcomes.

## Data Availability

The original contributions presented in the study are included in the article/Supplementary material, further inquiries can be directed to the corresponding author.
