# Supplementary material for: Climate change, pharmaceuticals and prescribing patterns: a scoping review and proposal for the MAMS model for healthcare educators
Source: Front Public Health. 2026 Aug 24;14:1806075. doi: 10.3389/fpubh.2026.1806075 (PMC13548501; doi:10.3389/fpubh.2026.1806075)
Supplement: Supplementary file 1 [file Data_Sheet_1.DOCX]

**Appendix A: Inclusion / Exclusion Criteria**

|  | ***Inclusion*** |
| --- | --- |
| Source Type and Data | English and Spanish language; Peer‑reviewed empirical studies of any methodology, including quantitative, qualitative, mixed‑methods, observational, experimental, quasi‑experimental, descriptive, and program‑evaluation designs. Included sources reported an implemented intervention involving health professionals or health‑professional learners and were identified through searches of six major bibliographic databases.  Publication date January 1, 1988 and April 1, 2025. |
| Healthcare Specialty / Target Audience | Studies involving healthcare professionals or trainees with prescriptive authority (e.g., physicians, nurse practitioners, physician assistants, clinical pharmacists with prescribing privileges, anesthesia, pre-med students) |
| Primary Clinical Domain | Must relate to at least one MAMS domain: |
|  | – Climate change impacts on health  – Pharmaceutical development, management, or utilization  – Prescribing patterns or medication-related decisions |
| Educational Model / Framework | Framework  **Definition:**  A *framework* is a **conceptual structure** that outlines key domains, competencies, principles, or components related to a topic, but **does not constitute a complete curriculum** on its own.  **Characteristics:**   - Conceptual, high-level - Organizes ideas or competencies - Not necessarily attached to delivery methods or assessments - Often used as a foundation for curriculum development |
|  | Curricula:  **Definition:**  A *curriculum* is a **structured, sequenced program of learning** designed to achieve specific competencies or learning outcomes over time. It typically includes learning objectives, content, teaching methods, assessment strategies, and sometimes implementation plans.  **Characteristics:**   - Multi-session or longitudinal - Formalized (institutional or organizational adoption) - Includes objectives and/or competencies - Assessment or evaluation plan may be present |
|  | Workshop (singular event)  **Definition:**  A *workshop* is a **short, focused educational session**, usually interactive, designed to build a specific skill or knowledge set.  **Characteristics:**   - Brief (1–4 hours, sometimes up to a full day) - Practical, applied learning - Often includes case discussions, role plays, active learning - Single event rather than longitudinal |
|  | Other  **Definition:** “Other” refers to any educational intervention, resource, or activity that supports learning but does not meet the defining characteristics of a curriculum, framework, or workshop. These may include standalone materials, brief learning tools, informal or ad‑hoc teaching activities, or educational components embedded within broader initiatives.  **Characteristics:**   - Does **not** meet criteria for a structured curriculum, conceptual framework, or interactive workshop - May be **informal**, **asynchronous**, **single‑component**, or **resource‑based** - Often focuses on **information dissemination** rather than competency-based learning |

**Use of Generative Artificial Intelligence in Drafting Operational Definitions for Educational Domains**

Generative artificial intelligence (ChatGPT, OpenAI GPT-5.1, accessed February 2026) was used to assist the research team in drafting operational definitions for educational categories relevant to the scoping review (e.g., curriculum, framework, workshop). The tool was not listed as an author, and the research team independently reviewed all AI-generated content for accuracy, consistency with the literature, and originality. The AI tool was not involved in data analysis, interpretation, or decision-making, and it was not listed as an author. All content was subsequently revised, validated, and integrated by the authors. AI outputs were limited to draft definitions for the educational categories. All final definitions were edited and validated by the authors, who retain full responsibility for the manuscript.

## **Prompts Provided to the AI**

## **Initial Prompt:** *“We are developing a scoping review model on climate change and prescribing and medication. We have reviewed a bunch of articles and are now in the building of a table. The articles must have discussed some form of education (development of a curriculum, framework, workshop, panel or some other form to discuss these concepts). How might you go about defining each of these in order for us to categorize our papers?”*

## **Final Prompt:** *“Can you tell me the exact prompts used to define this Operational Codebook for Categorizing Educational Elements?”*

- **Resulting Data:** Operational definitions of education domains, categorized in Table 1 for included studies
  - Framework
    - **Definition:**
       A *framework* is a **conceptual structure** that outlines key domains, competencies, principles, or components related to a topic, but **does not constitute a complete curriculum** on its own.
    - **Characteristics:**
      - Conceptual, high-level
      - Organizes ideas or competencies
      - Not necessarily attached to delivery methods or assessments
      - Often used as a foundation for curriculum development
  - Curricula:
    - **Definition:**
       A *curriculum* is a **structured, sequenced program of learning** designed to achieve specific competencies or learning outcomes over time. It typically includes learning objectives, content, teaching methods, assessment strategies, and sometimes implementation plans.
    - **Characteristics:**
      - Multi-session or longitudinal
      - Formalized (institutional or organizational adoption)
      - Includes objectives and/or competencies
      - Assessment or evaluation plan may be present
  - Workshop (singular event)
    - **Definition:**
       A *workshop* is a **short, focused educational session**, usually interactive, designed to build a specific skill or knowledge set.
    - **Characteristics:**
      - Brief (1–4 hours, sometimes up to a full day)
      - Practical, applied learning
      - Often includes case discussions, role plays, active learning
      - Single event rather than longitudinal
  - Other
    - **Definition:**
      “Other” refers to any educational intervention, resource, or activity that supports learning but does not meet the defining characteristics of a curriculum, framework, or workshop. These may include standalone materials, brief learning tools, informal or ad‑hoc teaching activities, or educational components embedded within broader initiatives.
    - **Characteristics:**
      - Does **not** meet criteria for a structured curriculum, conceptual framework, or interactive workshop
      - May be **informal**, **asynchronous**, **single‑component**, or **resource‑based**
      - Often focuses on **information dissemination** rather than competency-based learning

**Appendix B: Database Search Strategies**

**Searched December 1, 2023, rerun April 1, 2025, Firefox browser**

**Databases**

**CINAHL (EBSCOhost) mapping turned off
(MH "Health Personnel" OR pharmacist* OR pharmacy OR clinician* OR "mid-level practitioner" OR nurse* OR "health care professional*" OR "health profession*" OR "Health Personnel" OR "healthcare worker*" OR physician* OR doctor* OR osteopath* OR APRN* OR provider* OR "physician assistant*" OR "physician associate*" OR anesthetist* OR dentist* OR optometrist* OR podiatrist* OR psychiatrist* OR psychologist* OR "prescriptive authority") AND (MH "Prescriptive Authority" OR MH "Prescriptions, Non-Drug" OR MH "Prescriptions, Drug" OR MH "Pharmacogenetics" OR MH "Prescribing Patterns" OR pharmaceutic* OR medication* OR drug* OR agent* OR compound* OR medicine* OR "over the counter" OR prescription* OR prescrib* OR prescriptive OR dispens* OR pharmacology OR pharmacy OR dosage OR shelf-life OR "drug storage" OR pharmacogenomics OR pharmacodynamics OR pharmacokinetics OR pharmacotherapeutics OR "prescriptive authority" OR "practice authority" OR "scope of practice") AND (education* OR foundation* OR framework* OR model* OR competenc* OR domain* OR indicator* OR content OR intersection* OR inclusion OR relationship* OR curricul* OR student* OR aware* OR intervention*) AND (MH "Climate Change" OR "climate adapt*" OR "climate change*" OR "climate crisis" OR "climate disaster*" OR "climate emergen*" OR "climate related" OR "climate-related" OR "climate resilien*" OR "climate sensitive*" OR "climate warming" OR "climatic adapt*" OR "climatic change*" OR "climatic crisis" OR "climatic disaster*" OR "climatic emergen*" OR "climatic related" OR "climatic-related" OR "climatic resilien*" OR "climatic sensitiv*" OR "climatic warming" OR "climate model*" OR "climate predict*" OR "climate simulat*" OR "carbon offset*" OR "carbon sequestration*" OR "carbon sink*" OR "air quality" OR "atmospheric carbon dioxide" OR "atmospheric CO2" OR "CO2 level*" OR "increasing carbon dioxide" OR "increasing CO2" OR "extreme heat" OR "extreme temperature*" OR "heat wave*" OR heatwave* OR "hot temperature*" OR "extreme weather" OR "el nino" OR "la nina" OR "severe weather" OR "extreme hot weather" OR "extreme cold weather" OR "global temperature*" OR "global warming" OR "greenhouse effect*" OR "greenhouse gas*" OR "heat exhaustion" OR "heat-related illness*" OR "heat-related disorder*" OR "heat stress" OR "heat stroke" OR "arctic shrink*" OR "deglaciat*" OR "sea level*" OR "sea surface warming" OR "sea temperature*" OR "sun stroke" OR sunstroke OR "environmentally sustainab*" OR "environmental sustainab*" OR "climate change mitigation" OR "sustainable healthcare" OR "sustainable health care" OR "green practice*" OR "green principle*")**

**Limits: English, Spanish, 1988-2025, Human**

**Embase.com (Elsevier) mapping turned off
(pharmacist* OR pharmacy OR clinician* OR "mid-level practitioner" OR nurse* OR "health care professional*" OR "health profession*" OR "Health Personnel" OR "healthcare worker*" OR physician* OR doctor* OR osteopath* OR APRN* OR provider* OR "physician assistant*" OR "physician associate*" OR anesthetist* OR dentist* OR optometrist* OR podiatrist* OR psychiatrist* OR psychologist* OR "prescriptive authority"):ab,ti AND (pharmaceutic* OR medication* OR drug* OR agent* OR compound* OR medicine* OR "over the counter" OR prescription* OR prescrib* OR prescriptive OR dispens* OR pharmacology OR pharmacy OR dosage OR shelf-life OR "drug storage" OR pharmacogenomics OR pharmacodynamics OR pharmacokinetics OR pharmacotherapeutics OR "prescriptive authority" OR "practice authority" OR "scope of practice"):ab,ti AND (education* OR foundation* OR framework* OR model* OR competenc* OR domain* OR indicator* OR content OR intersection* OR inclusion OR relationship* OR curricul* OR student* OR aware* OR intervention*):ab,ti AND ("climate adapt*" OR "climate change*" OR "climate crisis" OR "climate disaster*" OR "climate emergen*" OR "climate related" OR "climate-related" OR "climate resilien*" OR "climate sensitive*" OR "climate warming" OR "climatic adapt*" OR "climatic change*" OR "climatic crisis" OR "climatic disaster*" OR "climatic emergen*" OR "climatic related" OR "climatic-related" OR "climatic resilien*" OR "climatic sensitiv*" OR "climatic warming" OR "climate model*" OR "climate predict*" OR "climate simulat*" OR "carbon offset*" OR "carbon sequestration*" OR "carbon sink*" OR "air quality" OR "atmospheric carbon dioxide" OR "atmospheric CO2" OR "CO2 level*" OR "increasing carbon dioxide" OR "increasing CO2" OR "extreme heat" OR "extreme temperature*" OR "heat wave*" OR heatwave* OR "hot temperature*" OR "extreme weather" OR "el nino" OR "la nina" OR "severe weather" OR "extreme hot weather" OR "extreme cold weather" OR "global temperature*" OR "global warming" OR "greenhouse effect*" OR "greenhouse gas*" OR "heat exhaustion" OR "heat-related illness*" OR "heat-related disorder*" OR "heat stress" OR "heat stroke" OR "arctic shrink*" OR "deglaciat*" OR "sea level*" OR "sea surface warming" OR "sea temperature*" OR "sun stroke" OR sunstroke OR "environmentally sustainab*" OR "environmental sustainab*" OR "climate change mitigation" OR "sustainable healthcare" OR "sustainable health care" OR "green practice*" OR "green principle*"):ab,ti**

**AND [1988-2025]/py AND ([english]/lim OR [spanish]/lim) AND [humans]/lim**

**ERIC (EBSCOhost) mapping turned off
(pharmacist* OR pharmacy OR clinician* OR "mid-level practitioner" OR nurse* OR "health care professional*" OR "health profession*" OR "Health Personnel" OR "healthcare worker*" OR physician* OR doctor* OR osteopath* OR APRN* OR provider* OR "physician assistant*" OR "physician associate*" OR anesthetist* OR dentist* OR optometrist* OR podiatrist* OR psychiatrist* OR psychologist* OR "prescriptive authority") AND (pharmaceutic* OR medication* OR drug* OR agent* OR compound* OR medicine* OR "over the counter" OR prescription* OR prescrib* OR prescriptive OR dispens* OR pharmacology OR pharmacy OR dosage OR shelf-life OR "drug storage" OR pharmacogenomics OR pharmacodynamics OR pharmacokinetics OR pharmacotherapeutics OR "prescriptive authority" OR "practice authority" OR "scope of practice") AND (education* OR foundation* OR framework* OR model* OR competenc* OR domain* OR indicator* OR content OR intersection* OR inclusion OR relationship* OR curricul* OR student* OR aware* OR intervention*) AND ("climate adapt*" OR "climate change*" OR "climate crisis" OR "climate disaster*" OR "climate emergen*" OR "climate related" OR "climate-related" OR "climate resilien*" OR "climate sensitive*" OR "climate warming" OR "climatic adapt*" OR "climatic change*" OR "climatic crisis" OR "climatic disaster*" OR "climatic emergen*" OR "climatic related" OR "climatic-related" OR "climatic resilien*" OR "climatic sensitiv*" OR "climatic warming" OR "climate model*" OR "climate predict*" OR "climate simulat*" OR "carbon offset*" OR "carbon sequestration*" OR "carbon sink*" OR "air quality" OR "atmospheric carbon dioxide" OR "atmospheric CO2" OR "CO2 level*" OR "increasing carbon dioxide" OR "increasing CO2" OR "extreme heat" OR "extreme temperature*" OR "heat wave*" OR heatwave* OR "hot temperature*" OR "extreme weather" OR "el nino" OR "la nina" OR "severe weather" OR "extreme hot weather" OR "extreme cold weather" OR "global temperature*" OR "global warming" OR "greenhouse effect*" OR "greenhouse gas*" OR "heat exhaustion" OR "heat-related illness*" OR "heat-related disorder*" OR "heat stress" OR "heat stroke" OR "arctic shrink*" OR "deglaciat*" OR "sea level*" OR "sea surface warming" OR "sea temperature*" OR "sun stroke" OR sunstroke OR "environmentally sustainab*" OR "environmental sustainab*" OR "climate change mitigation" OR "sustainable healthcare" OR "sustainable health care" OR "green practice*" OR "green principle*")**

**Limits: English, Spanish, 1988-2025**

**Health Source: Nursing/Academic (EBSCOhost) mapping turned off
(DE "MEDICAL personnel" OR pharmacist* OR pharmacy OR clinician* OR "mid-level practitioner" OR nurse* OR "health care professional*" OR "health profession*" OR "Health Personnel" OR "healthcare worker*" OR physician* OR doctor* OR osteopath* OR APRN* OR provider* OR "physician assistant*" OR "physician associate*" OR anesthetist* OR dentist* OR optometrist* OR podiatrist* OR psychiatrist* OR psychologist* OR "prescriptive authority") AND (DE "MEDICAL prescriptions" OR DE "PRESCRIPTION writing" OR pharmaceutic* OR medication* OR drug* OR agent* OR compound* OR medicine* OR "over the counter" OR prescription* OR prescrib* OR prescriptive OR dispens* OR pharmacology OR pharmacy OR dosage OR shelf-life OR "drug storage" OR pharmacogenomics OR pharmacodynamics OR pharmacokinetics OR pharmacotherapeutics OR "prescriptive authority" OR "practice authority" OR "scope of practice") AND (education* OR foundation* OR framework* OR model* OR competenc* OR domain* OR indicator* OR content OR intersection* OR inclusion OR relationship* OR curricul* OR student* OR aware* OR intervention*) AND (DE "Climate Change" OR "climate adapt*" OR "climate change*" OR "climate crisis" OR "climate disaster*" OR "climate emergen*" OR "climate related" OR "climate-related" OR "climate resilien*" OR "climate sensitive*" OR "climate warming" OR "climatic adapt*" OR "climatic change*" OR "climatic crisis" OR "climatic disaster*" OR "climatic emergen*" OR "climatic related" OR "climatic-related" OR "climatic resilien*" OR "climatic sensitiv*" OR "climatic warming" OR "climate model*" OR "climate predict*" OR "climate simulat*" OR "carbon offset*" OR "carbon sequestration*" OR "carbon sink*" OR "air quality" OR "atmospheric carbon dioxide" OR "atmospheric CO2" OR "CO2 level*" OR "increasing carbon dioxide" OR "increasing CO2" OR "extreme heat" OR "extreme temperature*" OR "heat wave*" OR heatwave* OR "hot temperature*" OR "extreme weather" OR "el nino" OR "la nina" OR "severe weather" OR "extreme hot weather" OR "extreme cold weather" OR "global temperature*" OR "global warming" OR "greenhouse effect*" OR "greenhouse gas*" OR "heat exhaustion" OR "heat-related illness*" OR "heat-related disorder*" OR "heat stress" OR "heat stroke" OR "arctic shrink*" OR "deglaciat*" OR "sea level*" OR "sea surface warming" OR "sea temperature*" OR "sun stroke" OR sunstroke OR "environmentally sustainab*" OR "environmental sustainab*" OR "climate change mitigation" OR "sustainable healthcare" OR "sustainable health care" OR "green practice*" OR "green principle*")**

**Limits: 1988-2025**

**PubMed
(pharmacist*[tw] OR “Pharmacy"[Mesh] OR pharmacy[tw] OR "Pharmacists"[Mesh] OR clinician*[tw] OR "mid-level practitioner"[tw] OR nurse*[tw] OR "Nurses"[Mesh] OR "health care professional*"[tw] OR "health profession*"[tw] OR "Health Personnel"[Mesh:NoExp] OR "healthcare worker*"[tw] OR physician*[tw] OR doctor*[tw] OR "Physicians"[Mesh] OR osteopath*[tw] OR APRN*[tw] OR provider*[tw] OR "physician assistant*"[tw] OR "Physician Assistants"[Mesh] OR "physician associate*"[tw] OR anesthetist*[tw] OR dentist*[tw] OR optometrist*[tw] OR podiatrist*[tw] OR psychiatrist*[tw] OR psychologist*[tw]) AND (pharmaceutic*[tiab] OR medication*[tiab] OR drug*[tiab] OR agent*[tiab] OR compound*[tiab] OR medicine*[tiab] OR "over the counter"[tiab] OR "Pharmaceutical Preparations"[Mesh] OR "Prescriptions"[Mesh] OR prescription*[tiab] OR prescriptive[tw] OR prescrib*[tiab] OR dispens*[tiab] OR "Practice Patterns, Pharmacists'"[Mesh] OR "Pharmacology"[Mesh] OR pharmacology[tiab] OR pharmacy[tiab] OR dosage[tiab] OR shelf-life[tiab] OR "drug storage"[tiab] OR pharmacogenomics[tiab] OR pharmacodynamics[tiab] OR pharmacokinetics[tiab] OR pharmacotherapeutics[tiab] OR "prescriptive authority"[tw] OR "practice authority"[tw] OR "scope of practice"[tw]) AND (education*[tw] OR "Education, Professional"[Mesh] OR foundation*[tw] OR framework*[tw] OR model*[tw] OR competenc*[tw] OR domain*[tw] OR indicator*[tw] OR content[tiab] OR intersection*[tw] OR inclusion[tw] OR relationship*[tw] OR curricul*[tw] OR "Curriculum"[Mesh] OR student*[tiab] OR aware*[tiab] OR intervention*[tiab]) AND ("Climate Change"[mesh] OR "climate adapt*"[tw] OR "climate change*"[tw] OR "climate crisis"[tw] OR "climate disaster*"[tw] OR "climate emergen*"[tw] OR "climate related"[tw] OR "climate-related"[tw] OR "climate resilien*"[tw] OR "climate sensitive*"[tw] OR "climate warming"[tw] OR "climatic adapt*"[tw] OR "climatic change*"[tw] OR "climatic crisis"[tw] OR "climatic disaster*"[tw] OR "climatic emergen*"[tw] OR "climatic related"[tw] OR "climatic-related"[tw] OR "climatic resilien*"[tw] OR "climatic sensitiv*"[tw] OR "climatic warming"[tw] OR "Climate Models"[mesh] OR "climate model*"[tw] OR "climate predict*"[tw] OR "climate simulat*"[tw] OR "Carbon Sequestration"[mesh] OR "carbon offset*"[tw] OR "carbon sequestration*"[tw] OR "carbon sink*"[tw] OR "air quality"[tw] OR "atmospheric carbon dioxide"[tw] OR "atmospheric CO2"[tw] OR "Carbon Dioxide"[Mesh] OR "CO2 level*"[tw] OR "increasing carbon dioxide"[tw] OR "increasing CO2"[tw] OR "Extreme Heat"[mesh] OR "extreme heat"[tw] OR "extreme temperature*"[tw] OR "heat wave*"[tw] OR heatwave*[tw] OR "Hot Temperature"[Mesh] OR "hot temperature*"[tw] OR "Extreme Weather"[mesh] OR "extreme weather"[tw] OR "El Nino-Southern Oscillation"[mesh] OR "el nino"[tw] OR "la nina"[tw] OR "severe weather"[tw] OR "extreme hot weather"[tw] OR "extreme cold weather"[tw] OR "Global Warming"[mesh] OR "global temperature*"[tw] OR "global warming"[tw] OR "Greenhouse Effect"[mesh] OR "greenhouse effect*"[tw] OR "Greenhouse Gases"[mesh] OR "greenhouse gas*"[tw] OR "heat exhaustion"[tw] OR "heat-related illness*"[tw] OR "heat-related disorder*"[tw] OR "Heat Stress Disorders"[Mesh] OR "heat stress"[tw] OR "heat stroke"[tw] OR "Ice Cover"[mesh] OR "arctic shrink*"[tw] OR "deglaciat*"[tw] OR "Sea Level Rise"[mesh] OR "sea level*"[tw] OR "sea surface warming"[tw] OR "sea temperature*"[tw] OR "sun stroke"[tw] OR sunstroke[tw] OR "environmentally sustainab*"[tw] OR "environmental sustainab*"[tw] OR "climate change mitigation"[tw] OR "sustainable healthcare"[tw] OR "sustainable health care"[tw] OR "green practice*"[tw] OR "green principle*"[tw]) AND (english[Filter] OR spanish[Filter]) AND ("1988/01/01"[PDAT] : "2025/12/31"[PDAT]) NOT (("animals"[MeSH Terms] NOT "humans"[MeSH Terms]) OR "animal model*"[tiab])**

**Web of Science Core Collection (Clarivate) (Science Citation Index Expanded, Social Sciences Citation Index, Arts & Humanities Citation Index, Conference Proceedings Citation Index-Science, Conference Proceedings Citation Index-Social Sciences & Humanities, Book Citation Index– Science, Book Citation Index– Social Sciences & Humanities, Emerging Sources Citation Index, Current Chemical Reactions, Index Chemicus)
TS=(pharmacist* OR pharmacy OR clinician* OR "mid-level practitioner" OR nurse* OR "health care professional*" OR "health profession*" OR "Health Personnel" OR "healthcare worker*" OR physician* OR doctor* OR osteopath* OR APRN* OR provider* OR "physician assistant*" OR "physician associate*" OR anesthetist* OR dentist* OR optometrist* OR podiatrist* OR psychiatrist* OR psychologist* OR "prescriptive authority") AND TS=(pharmaceutic* OR medication* OR drug* OR agent* OR compound* OR medicine* OR "over the counter" OR prescription* OR prescrib* OR prescriptive OR dispens* OR pharmacology OR pharmacy OR dosage OR shelf-life OR "drug storage" OR pharmacogenomics OR pharmacodynamics OR pharmacokinetics OR pharmacotherapeutics OR "prescriptive authority" OR "practice authority" OR "scope of practice") AND TS=(education* OR foundation* OR framework* OR model* OR competenc* OR domain* OR indicator* OR content OR intersection* OR inclusion OR relationship* OR curricul* OR student* OR aware* OR intervention*) AND TS=("climate adapt*" OR "climate change*" OR "climate crisis" OR "climate disaster*" OR "climate emergen*" OR "climate related" OR "climate-related" OR "climate resilien*" OR "climate sensitive*" OR "climate warming" OR "climatic adapt*" OR "climatic change*" OR "climatic crisis" OR "climatic disaster*" OR "climatic emergen*" OR "climatic related" OR "climatic-related" OR "climatic resilien*" OR "climatic sensitiv*" OR "climatic warming" OR "climate model*" OR "climate predict*" OR "climate simulat*" OR "carbon offset*" OR "carbon sequestration*" OR "carbon sink*" OR "air quality" OR "atmospheric carbon dioxide" OR "atmospheric CO2" OR "CO2 level*" OR "increasing carbon dioxide" OR "increasing CO2" OR "extreme heat" OR "extreme temperature*" OR "heat wave*" OR heatwave* OR "hot temperature*" OR "extreme weather" OR "el nino" OR "la nina" OR "severe weather" OR "extreme hot weather" OR "extreme cold weather" OR "global temperature*" OR "global warming" OR "greenhouse effect*" OR "greenhouse gas*" OR "heat exhaustion" OR "heat-related illness*" OR "heat-related disorder*" OR "heat stress" OR "heat stroke" OR "arctic shrink*" OR "deglaciat*" OR "sea level*" OR "sea surface warming" OR "sea temperature*" OR "sun stroke" OR sunstroke OR "environmentally sustainab*" OR "environmental sustainab*" OR "climate change mitigation" OR "sustainable healthcare" OR "sustainable health care" OR "green practice*" OR "green principle*")**

**Limits: English, Spanish, 1988-2025**
